# Evaluation of immunogenicity-induced DNA vaccines against different SARS-CoV-2 variants

**DOI:** 10.1371/journal.pone.0295594

**Published:** 2023-12-07

**Authors:** Se Eun Kim, So Hee Park, Woo-Jung Park, Gayeong Kim, Seo Yeon Kim, Hyeran Won, Yun-Ho Hwang, Heeji Lim, Hyeon Guk Kim, You-Jin Kim, Dokeun Kim, Jung-Ah Lee

**Affiliations:** National Institute of Infectious Disease, National Institute of Health, Korea Disease Control and Prevention Agency, CheongJu, Chungcheongbuk-do, Republic of Korea; Fundacao Oswaldo Cruz - Instituto Rene Rachou, BRAZIL

## Abstract

Severe acute respiratory syndrome coronavirus 2 (SARS-CoV-2) emerged in 2019 and caused the coronavirus disease 2019 (COVID-19) pandemic worldwide. As of September 2023, the number of confirmed coronavirus cases has reached over 770 million and caused nearly 7 million deaths. The World Health Organization assigned and informed the characterization of variants of concern (VOCs) to help control the COVID-19 pandemic through global monitoring of circulating viruses. Although many vaccines have been proposed, developing an effective vaccine against variants is still essential to reach the endemic stage of COVID-19. We designed five DNA vaccine candidates composed of the first isolated genotype and major SARS-CoV-2 strains from isolated Korean patients classified as VOCs, such as Alpha, Beta, Gamma, and Delta. To evaluate the immunogenicity of each genotype via homologous and heterologous vaccination, mice were immunized twice within a 3-week interval, and the blood and spleen were collected 1 week after the final vaccination to analyze the immune responses. The group vaccinated with DNA vaccine candidates based on the S genotype and the Alpha and Beta variants elicited both humoral and cellular immune responses, with higher total IgG levels and neutralizing antibody responses than the other groups. In particular, the vaccine candidate based on the Alpha variant induced a highly diverse cytokine response. Additionally, we found that the group subjected to homologous vaccination with the S genotype and heterologous vaccination with S/Alpha induced high total IgG levels and a neutralization antibody response. Homologous vaccination with the S genotype and heterologous vaccination with S/Alpha and S/Beta significantly induced IFN-γ immune responses. The immunogenicity after homologous vaccination with S and Alpha and heterologous vaccination with the S/Alpha candidate was better than that of the other groups, indicating the potential for developing novel DNA vaccines against different SARS-CoV-2 variants.

## Introduction

Severe acute respiratory syndrome coronavirus 2 (SARS-CoV-2), first identified and isolated in Wuhan, China in late 2019, has spread worldwide, with more than 770 million confirmed coronavirus cases and nearly 7 million deaths, according to the World Health Organization (WHO) Coronavirus Disease 2019 (COVID-19) Dashboard as of September 13, 2023 [1]. The most prevalent symptoms of SARS-CoV-2 infection are fever, dry cough, fatigue, myalgia, dyspnea, anorexia, and acute pneumonia [2]. SARS-CoV-2 has mutated over time, resulting in new variants such as Alpha, Beta, Gamma, and Delta. The world has faced a high burden of COVID-19, owing to the risk of reinfection even after vaccination [3]. Several reports have shown that receiving a single vaccine dose can rapidly induce cellular and humoral immune responses, but it is not enough to maintain long-term immunity [4]. Moreover, with the emergence of new variants, people face an increased risk of breakthrough reinfections and spreading the virus to others despite full or partial vaccination and prior infection with SARS-CoV-2 [5]. Previous studies have demonstrated that obtaining a second booster is essential for preventing the natural decline in anti-SARS-CoV-2 antibody levels and neutralizing activity over time from a single vaccine dose [6].

The emergence of new variants poses a risk; even full vaccination has resulted in a decline in the effectiveness of current vaccines, such as Moderna and Pfizer-BioNTech, against new variant infections based on the S genotype, the first isolated and circulating virus [7]. Although several effective COVID-19 vaccine platforms based on the S genotype have been proposed, developing safe and novel vaccine strategies is still a high priority to reduce the risk of reinfection and severe disease leading to death caused by new variants [8].

Our previous study on DNA vaccine candidates presents a safe platform technology for development in an emergency such as the COVID-19 pandemic. It demonstrates the superiority of various vaccine candidates, including the entire spike protein S genotype that includes the first virus strain (Wuhan strain) isolated in Korea, to truncated variants by showing vaccine-induced immunity. Additionally, their good safety profile and stability at room temperature makes DNA vaccine platforms more convenient to store and ship than other platforms [9,10]. However, DNA vaccines often do not induce significant clinical benefits despite eliciting strong humoral and cellular immune responses [10,11]. In order to address these constraints, several strategies, such as prime-boosting immunization and electroporation, can improve the effectiveness of the delivery methods by inducing the activation of immune response [12,13]. Moreover, to further boost vaccine strategies to improve the immune response after vaccination, research on homologous and heterologous prime-boost vaccination has gained great interest worldwide. Previous research on homologous and heterologous vaccination against SARS-CoV-2 and influenza indicated that the level of humoral and cellular immunity was successfully elevated by increasing neutralization activity and inducing T cell responses [14]. Therefore, we combined DNA vaccination and electroporation via intramuscular injection in a platform to follow a prime-boost regimen and constructed five DNA vaccine candidates based on the major SARS-CoV-2 strains, including the first genotype S, and Alpha, Beta, Gamma, and Delta, which are classified as VOCs by the WHO. We also evaluated the humoral and cellular immune responses in mice vaccinated with these DNA vaccine candidates to assess their immunogenicity.

## Materials and methods

### Cells and viruses

Vero E6 cells were passaged in Dulbecco’s modified Eagle’s medium (Gibco, USA) supplemented with 10% heat-inactivated fetal bovine serum (Gibco, USA) containing 1% penicillin/streptomycin (Gibco, USA) and maintained in a humidified 5% CO_2_ incubator at 37°C. SARS-CoV-2 strains, including the S, Alpha, Beta, Gamma, and Delta variants (NCCP43326, NCCP43381, NCCP43382, NCCP43388, NCCP43390) used in this study as infectious viruses, were collected from human isolates from Korea and provided by the National Culture Collection for Pathogens (NCCP). All sequences were deposited in GenBank under the accession numbers MW46691.1, OL958605.1, OL958606.1, OL958675.1, and OL966996.1. These strains were passaged and titrated via plaque-forming units in cells. All experiments with live viruses were performed in a biosafety level 3 (BL3) facility, following all standard precautions.

### COVID-19 DNA vaccine construction and expression

Gene sequences encoding the spike proteins of isolates from Korean patients belonging to the S genotype and the Alpha, Beta, Gamma, and Delta variants were aligned, and consensus sequences from 63, 98, 73, 17, and 536 isolates, respectively, were determined from the Global Initiative for Sharing All Influenza Data using BioEdit (Informer Technologies, Inc., USA). The sequences of the vaccine candidates were optimized using the OptimumGene^TM^ algorithm to increase their expression and were synthesized by GenScript Biotech (USA). The synthetic full-length S protein of the five strains was subcloned into the mammalian expression vector pVax1 (Invitrogen, USA) and an N-terminal tPA leader sequence was added to enhance *in vivo* expression. Chemically competent cells were prepared using the *Escherichia coli* JM108 strain. *E*. *coli* were incubated along with the plasmids at 37°C and harvested. The plasmids were purified and subjected to the endotoxin test. The endotoxin levels of the DNA vaccine were found to be 0.01 EU/μg. The recombinant plasmid was purified using an EndoFree Plasmid Giga Kit (QIAGEN GmbH, China) and stored at −20°C until use.

### Mouse experiments

The mouse studies were approved by the Institutional Animal Care and Use Committee of the Korea Disease Control and Prevention Agency (KDCA-IACUC-21-037). Briefly, 4-week-old female C57BL/6 mice were homologously or heterologously immunized twice with 50 μg of the DNA vaccine candidate based on diverse genotypes at 3-week intervals. After intramuscular immunization with a single dose of the DNA vaccine, a two-needle array electrode pair was immediately inserted into the tibialis anterior muscle of the mice. After delivering three 100 V-pulses using an ECM 830 square and an electrophoresis system (BTX, USA), the needles were carefully checked for proper contact between the target tissue and electrode. To evaluate the humoral immune responses, the sera of the mice were collected 4 weeks after the first vaccine dose was administered.

### Enzyme-linked immunosorbent assay (ELISA)

To analyze the levels of SARS-CoV-2-specific total IgG, each well of Nunc MaxiSorp 96-well plates (Thermo Scientific, USA) was coated with 50 ng of SARS-CoV-2 S1+S2 ECD protein (Sino Biological, China) and incubated overnight at 4°C. After removing the proteins, the wells were blocked with 1% bovine serum albumin in phosphate-buffered saline (PBS) for 2 h at 37°C and washed twice with washing buffer containing PBS with 0.02% Tween-20 (0.02% PBST). The immunized mouse sera were serially diluted 2-fold, added to the plates, and incubated for 1 h at 37°C. After washing thrice with 0.02% PBST, horseradish peroxidase (HRP)-conjugated anti-mouse IgG (Invitrogen, USA) was added to the wells and incubated at 37°C for 1 h. Next, the wells were washed five times with 0.02% PBST. Tetramethyl benzidine (TMB) substrate (Thermo Fisher Scientific, USA) was then added to the wells and incubated with the HRP-conjugated anti-mouse IgG secondary antibody for 10 min at 20–25°C. Subsequently, the reaction between TMB and HRP-conjugated anti-mouse IgG was stopped by adding the corresponding stop solution. The absorbance of the contents of the wells was recorded at 450 nm using a Spectra Max i3X microplate reader (Molecular Devices, USA). The results of half-maximal effective concentration (EC_50_) were generated by a four-parameter logistic function [15].

To evaluate the SARS-CoV-2-specific IgG isotypes, the protocols employed were similar to those of ELISA for total IgG without the secondary antibodies, which were HRP-conjugated goat anti-mouse IgG1, IgG2a, IgG2b, IgG2c, and IgG3 (Invitrogen, USA).

### Viral neutralization assay

The plaque reduction neutralization test (PRNT) and surrogate virus neutralization test (sVNT) were performed to evaluate viral neutralization. For PRNT, 12-well plates seeded with 2 × 10^5^ Vero E6 cells in triplicate were incubated overnight at 37°C in a 5% CO_2_ incubator. SARS-CoV-2 virus particles (60 plaque-forming units) were mixed with an equal volume of 2-fold serial dilutions of heat-inactivated immunized mouse sera and incubated at 37°C for 1 h. Then, the serum-virus mixtures were added to the cells in the prepared 12-well plates and incubated for 1 h. The infected cells were then overlaid with 1% agarose (Lonza, Switzerland) in 2× Minimum Essential Medium (Gibco, USA) containing 4% heat-inactivated fetal bovine serum (Gibco, USA) and incubated at 37°C for 3 days. Then, the cells were stained with 0.8% crystal violet solution after fixation with 10% formaldehyde. After removal and drying at room temperature, the plaques were counted immediately. The neutralizing antibody titer was defined as the dilution factor corresponding to a 50% plaque reduction compared with the control. Based on the Kärber formula, Neutralizing antibody titer (NT titer) was calculated for the PRNT titer [16].

For sVNT, the sera of vaccinated mice were mixed with a diluted HRP-receptor binding domain solution in a 1:1 volume ratio, and the mixtures were incubated at 37°C for 30 min. The sample mixture in duplicate was added to 96-well plates from a commercial kit (GenScript Biotech, USA) and incubated at 37°C for 15 min. After washing the plates using a Biotek 405 TS microplate washer (Agilent, USA), a TMB solution was added to the wells and reacted for 15 min at 25°C, and the corresponding stop solution was added to stop the reaction. Finally, the absorbance of the wells was recorded at 450 nm on a Spectra Max i3X spectrophotometer (Molecular Devices, USA).

### Cytokine analysis

To evaluate interferon-gamma (IFN-γ)-secreting splenocytes, we performed an enzyme-linked immunosorbent spot (ELISpot) assay using a Mouse IFN-γ ELISpot kit (R&D Systems, USA). Briefly, 5 × 10^5^ splenocytes from immunized mice were seeded in 96-well polyvinylidene fluoride-backed microplates and cultured in RPMI 1640 medium (Gibco, USA) stimulated with 1 μg of a SARS-CoV-2 spike glycoprotein peptide pool (GenScript Biotech, USA) overnight at 37°C. The peptide pool used in this study included 315 peptides through the entire spike protein of SARS-CoV-2 (15-mers with 11 amino acid overlaps). The plates were washed after stimulation and incubated at 20–25°C for 2 h with biotinylated anti-IFN-γ antibodies. Alkaline phosphatase-conjugated streptavidin was then added to each well and incubated at room temperature for 2 h. Spot development was assessed after adding a 3-amino-9-ethylcarbazole chromogen solution for 20 min, and the number of colored spots formed was counted using a CTL Immunospot reader (Immunospot, USA). To analyze cytokine expression, the MILLIPLEX Mouse High Sensitivity T Cell Magnetic Bead Panel kit (Millipore, USA) was used according to the manufacturer’s instructions and the samples were subjected to a Luminex MAGPIX system (Luminex Corp., USA).

### Statistical analysis

Statistical analysis was performed using one-way ANOVA with Dunnett’s and Tukey’s multiple comparison tests, one-way ANOVA with uncorrected Fisher’s LSD test, and two-way ANOVA with Dunnett’s and Tukey’s multiple comparison tests. The statistical tests were adjusted for multiple comparisons between the control group vaccinated with pVax1. All statistical analyses were conducted using the GraphPad Prism software (La Jolla, USA). *P-*values < 0.05 were defined as statistically significant.

## Results

### Design of DNA vaccine candidates against SARS-CoV-2

**Fig 1:**
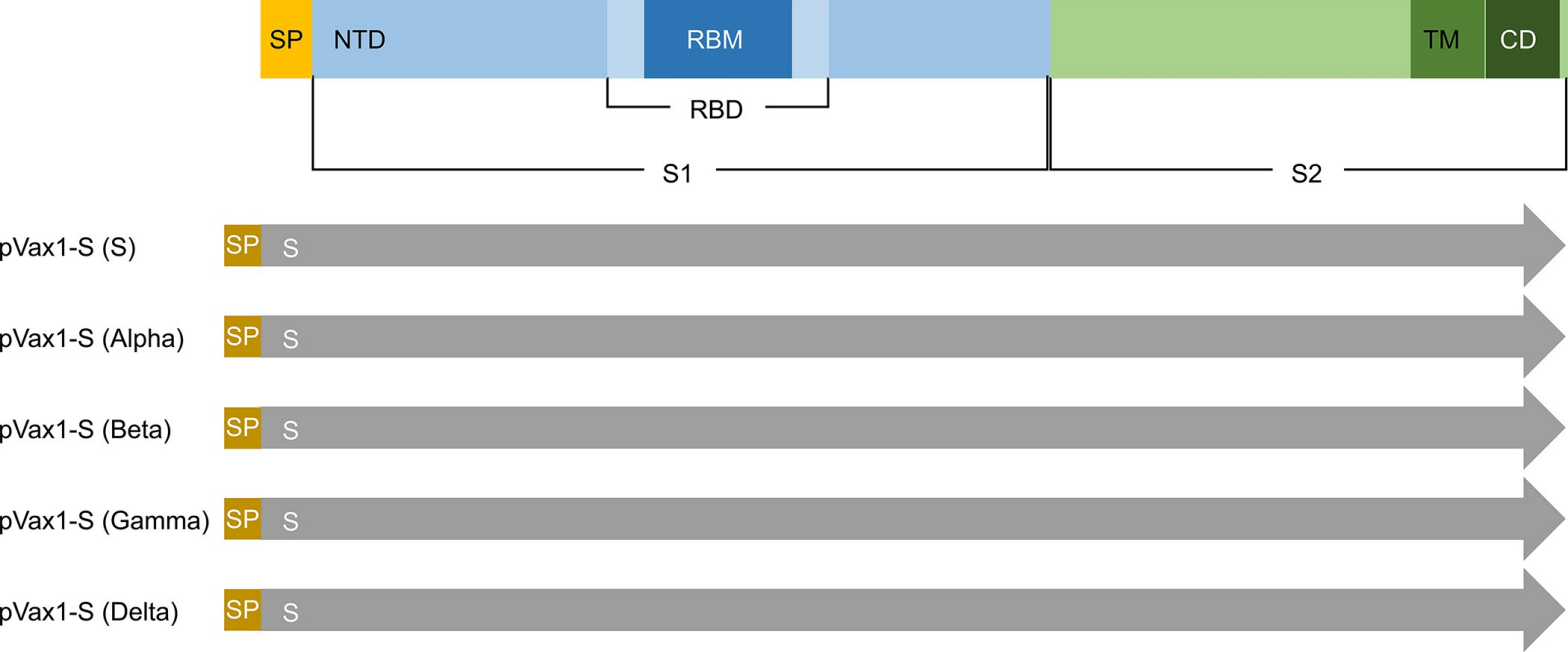
Design of severe acute respiratory syndrome coronavirus 2 (SARS-CoV-2) DNA vaccine candidates. Schematic diagram of the five DNA vaccine constructs used in this study. Five DNA vaccine candidates were designed using various SARS-CoV-2 strains, including the first genotype S and the Alpha, Beta, Gamma, and Delta variants, which are classified as variants of concern (VOCs) by the World Health Organization. Abbreviations: CD, cytoplasmic domain; NTD, N-terminal domain; RBD, receptor-binding domain; RBM: Receptor-binding motif; SP: Signal peptide; TM: Transmembrane domain.

We constructed five DNA vaccine candidates expressing the full-length S protein of five genotypes: the S genotype and the Alpha, Beta, Gamma, and Delta variants (Fig 1). The DNA sequences of all five SARS-CoV-2 genotypes were aligned with isolated and reported sequences from Korean patients at the Global Initiative for Sharing All Influenza Data using 63 isolates of the S genotype, 98 isolates of Alpha, 73 isolates of Beta, 16 isolates of Gamma, and 537 isolates of Delta. The tPA leader sequence was linked to the DNA sequences of the spike protein, and the full DNA vaccine sequences were optimized to enhance expression and immunogenicity. The optimized sequence was digested with *Bam*HI and *Xho*I and cloned into the pVax1 vector for expression.

### Immune response induced by SARS-CoV-2 DNA vaccine candidates in mice

**Fig 2:**
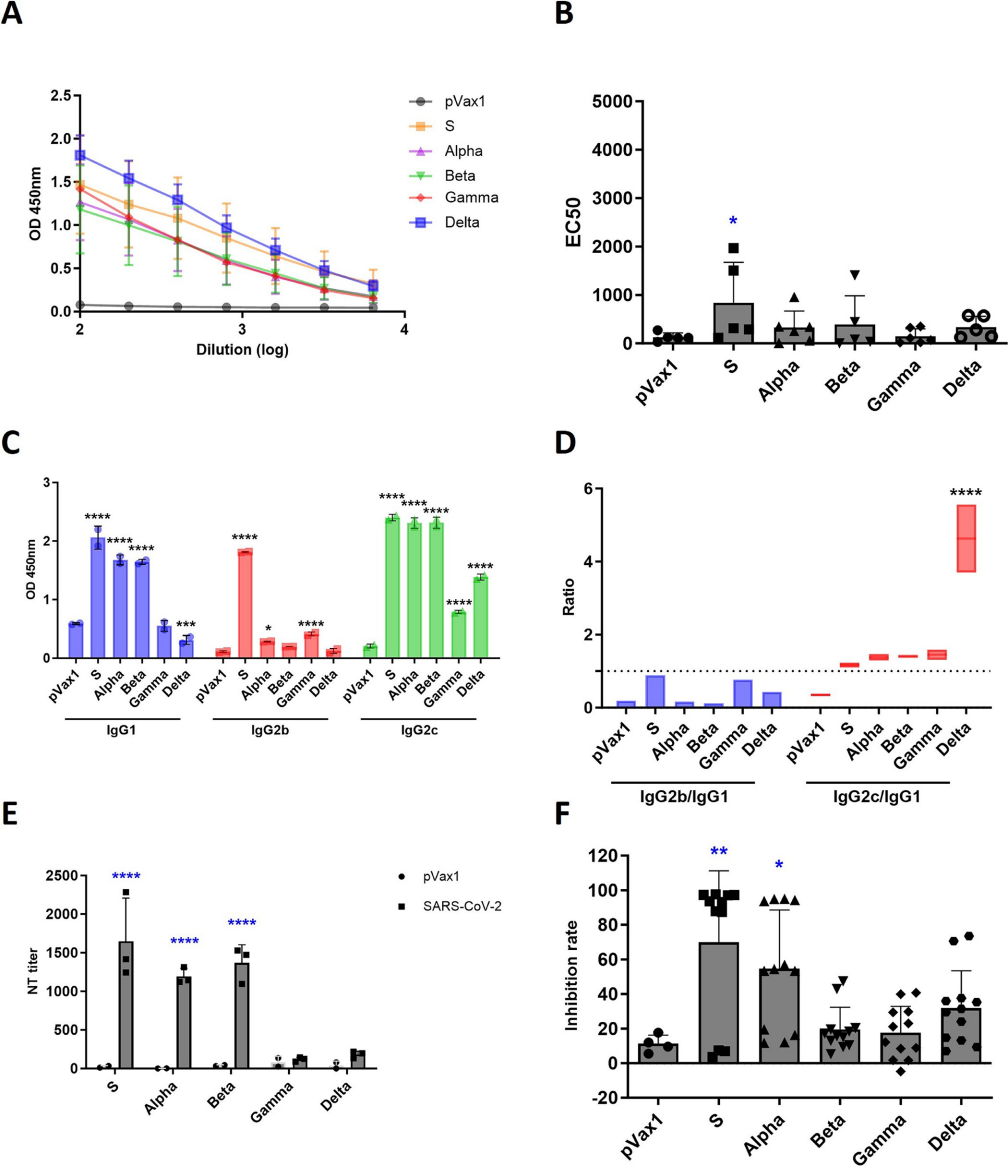
Evaluating humoral immunogenicity induced by severe acute respiratory syndrome coronavirus 2 (SARS-CoV-2) DNA vaccine candidates. (A) The blood samples of vaccinated mice were collected one week after the final vaccination. Sera of vaccinated mice were analyzed for SARS-CoV-2 specific total IgG using ELISA. Each sample was tested in duplicate. (B) The total IgG titers were calculated as EC50 (half maximal effective concentration). *P*-values were determined using one-way ANOVA with uncorrected Fisher’s LSD test. (C) The IgG subtypes in the sera of vaccinated mice were also analyzed using ELISA. *P*-values were determined using two-way ANOVA with Tukey’s test. (D) The ratios of IgG2b to IgG1 and IgG2c to IgG1 were calculated. *P*-values were determined using two-way ANOVA with Tukey’s test. (E) The neutralizing antibody titers in the sera of the vaccinated mice were analyzed via a plaque reduction neutralization test (PRNT) using the SARS-CoV-2 virus. *P*-values were determined using two-way ANOVA with Tukey’s test. (F) The sera of mice were tested for their neutralization rate using a SARS-CoV-2 surrogate virus neutralization test (sVNT). *P*-values were determined using one-way ANOVA with Dunnett’s test. * *P* < 0.05, ** *P* < 0.01, *** *P* < 0.001, **** *P* < 0.0001.

**Fig 3:**
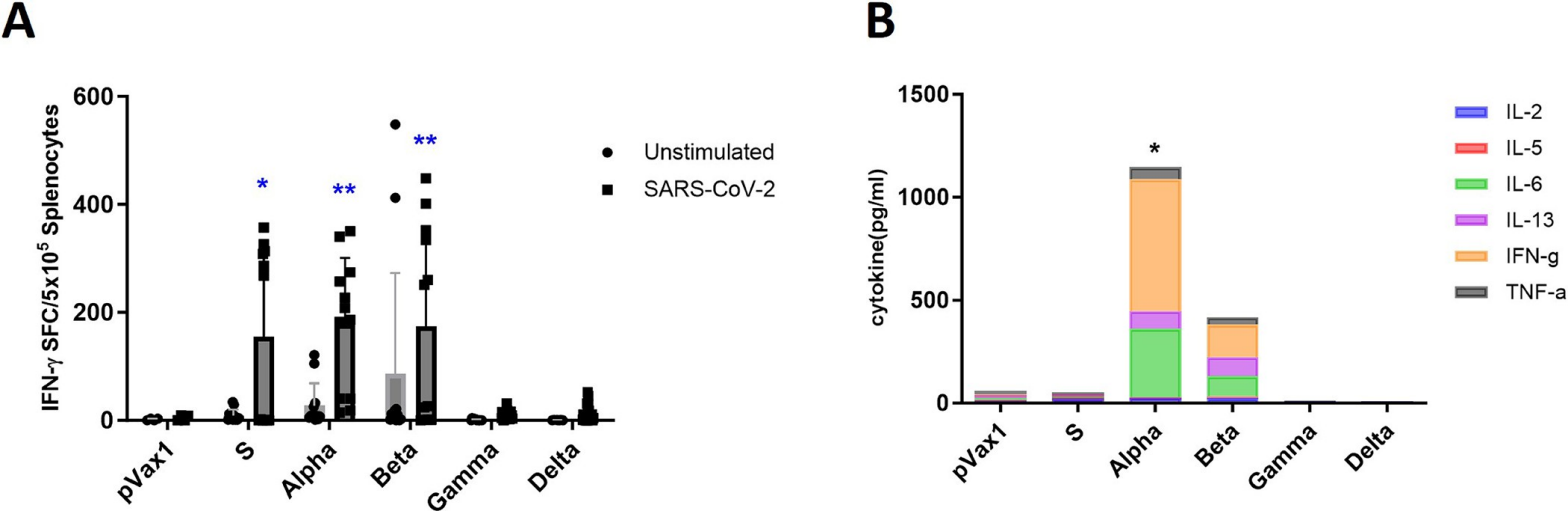
Induction of cell-mediated immune responses in mice after immunization with DNA vaccine candidates. (A) Splenocytes from mice immunized with the DNA vaccine candidates were collected 1 week after the final vaccination. They were tested in duplicate using a spike glycoprotein peptide pool and compared to those in unstimulated cells. Sample wells with spots were measured using a CTL Immunospot reader. Data are shown as the mean ± SD from individual mice (n = 6) per group. *P*-values were determined using two-way ANOVA with Dunnett’s test. (B) Multiplex cytokines using the supernatant of a mouse splenocyte culture were measured using a Luminex Megapix system. *P*-values were determined using two-way ANOVA with Tukey’s post-hoc test. * *P* < 0.05, ** *P* < 0.01.

To evaluate the immune response induced by the vaccine candidates in mice, C57BL/6 mice were randomly divided into seven groups (n = 6 mice per group) and vaccinated via electroporation with 50 μg of the DNA vaccine candidates twice at 3-week intervals. The mouse sera were collected one week after the final vaccine administration to analyze spike-specific IgG levels via ELISA and neutralizing antibody responses. Mice immunized with the DNA vaccine candidates based on the S genotype and Alpha and Beta variants had elevated SARS-CoV-2-specific total IgG levels compared with those of the other groups (Fig 2A and 2B). The IgG subtypes from vaccinated mice indicated that higher IgG1 and IgG2c levels were produced than those of the other IgG subtypes in all groups (Fig 2C and 2D). In particular, the S genotype-vaccinated group showed a high IgG2b subtype immune response. To quantify the neutralizing antibodies against SARS-CoV-2 *in vitro*, PRNT using a mixture of diluted serum samples containing viruses was performed in triplicate and sVNT was performed by mixing the mouse sera with a diluted HRP-receptor binding domain solution in duplicate. The level of neutralizing antibodies increased in the groups vaccinated with the candidates based on the S genotype and Alpha and Beta variants compared with the Gamma and Delta variants (Fig 2E). The S and Alpha groups showed higher inhibition rates than the other groups (Fig 2F). We also performed an ELISpot assay using vaccinated mouse splenocytes stimulated with a SARS-CoV-2 spike glycoprotein peptide pool and a multiplex cytokine assay to measure the level of antigen-specific T cell response induced by the DNA vaccine candidates. The S, Alpha, and Beta groups showed quantitatively more robust SARS-CoV-2-specific T cell responses than the Gamma and Delta groups (Fig 3A). In particular, the vaccine candidates based on the Alpha and Beta variants induced strong, diverse cytokine responses, showing significantly higher levels of IL-6, IL-13, and IFN-γ than the other candidates (Fig 3B). These data demonstrate that the mice vaccinated with the DNA vaccine candidates based on the S genotype and the Alpha and Beta variants elicited stronger humoral and cell-mediated immune responses than the mice administered with the other vaccine candidates.

### Immune responses in mice after cross-vaccination with SARS-CoV-2 DNA vaccine candidates

**Fig 4:**
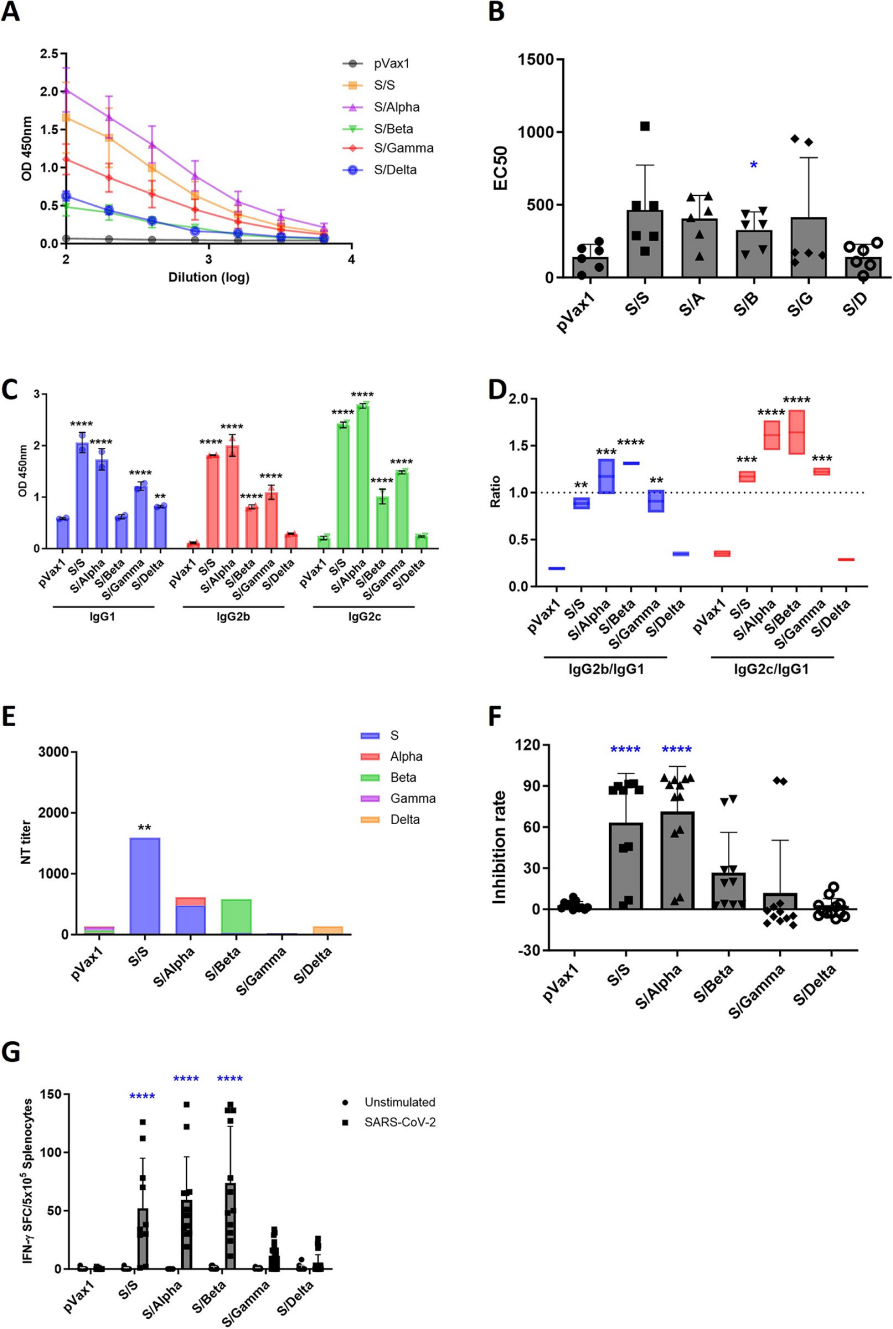
Evaluating the immunogenicity of cross-vaccination using severe acute respiratory syndrome coronavirus 2 (SARS-CoV-2) DNA vaccine candidates. (A) The blood samples of vaccinated mice were collected one week after the final vaccination, at week four after first vaccination. SARS-CoV-2-specific total IgG levels in the vaccinated mice were measured using ELISA. Each sample was tested in duplicate. (B) The total IgG titers were calculated as EC50 (half maximal effective concentration). *P*-values were determined using one-way ANOVA with Dunnett’s test. (C) The IgG subtypes in the sera of the vaccinated mice were also measured using ELISA. *P*-values were determined using two-way ANOVA with Dunnett’s test. (D) The ratios of IgG2b to IgG1 and IgG2c to IgG1 were calculated. *P*-values were determined using two-way ANOVA with Tukey’s test. (E) Neutralizing antibody titers in the vaccinated mouse sera were detected using a plaque reduction neutralization test (PRNT). *P*-values were determined using two-way ANOVA with Dunnett’s test. (F) Neutralizing antibody levels in the sera of the mice were tested using a SARS-CoV-2 surrogate virus neutralization test (sVNT) kit. *P*-values were determined using one-way ANOVA with Dunnett’s test. (G) An autopsy of the mice was performed one week after the final vaccination. The splenocytes of the sacrificed mice were isolated and the levels of cytokines secreted were measured using ELISpot. The splenocytes were stimulated with a SARS-CoV-2 spike glycoprotein peptide pool to determine if there was a significant difference compared to the unstimulated group, and the spots were counted using a CTL Immunospot reader. Data are shown as the means ± SD from individual mice (n = 6) per group. *P*-values were determined using two-way ANOVA with Tukey’s test. * *P* < 0.05, ** *P* < 0.01, *** *P* < 0.001, **** *P* < 0.0001.

To evaluate the immunogenicity elicited by the DNA vaccination regimens based on the SARS-CoV-2 variants, C57BL/6 mice were immunized twice at three-week intervals using five DNA vaccine candidates via homologous or heterologous vaccination and sacrificed one week after the final vaccination. Humoral immune responses, including IgG levels and neutralizing antibody titers, were analyzed using ELISA, PRNT, and sVNT assays. The mice in the S/S and S/Alpha groups showed high levels of SARS-CoV-2-specific total IgG and IgG subtypes, especially IgG1, IgG2b, and IgG2c (Fig 4A–4D). In addition, the S/S and S/Alpha groups showed high levels of neutralizing antibody responses (Fig 4E and 4F). Cellular immune response was also measured by determining the number of IFN-γ-secreting cells using an ELISpot assay. The mice vaccinated with S/Alpha and S/Beta showed robust IFN-γ immune responses compared to those in the S/Gamma and S/Delta groups (Fig 4G). These data indicated that homologous vaccination with the S genotype and heterologous vaccination with S/Alpha induced humoral and cellular immune responses in mice.

## Discussion

The emergence of SARS-CoV-2 in late 2019 has changed many aspects of human life, including the lockdown of economies, social disruption, public health, and human psychological states [17]. Therefore, developing a safe and effective vaccination strategy is the key to preventing increasing cases of hospitalization and death from SARS-CoV-2 infection.

Previous studies have provided various opinions on optimizing strategies using homologous and heterologous vaccinations against many diseases [18]. Several previous studies have indicated that numerous successful homologous vaccination strategies, such as the oral polio vaccine, hepatitis A and B vaccines, and *Haemophilus influenzae* type B vaccine, have been rigorously evaluated based on scientific evidence and clinical trials [18]. Moreover, various scientific studies confirm the beneficial effects of heterologous vaccination strategies against malaria, influenza, and human papillomavirus using different vaccine platforms, such as DNA, adenoviral vectors, modified vaccinia Ankara viral vectors, and recombinant subunit vaccine immunization regimens. These strategies have shown significantly increased humoral and cellular immunity [19–22]. Utilizing different vaccination strategies in advanced vaccine development plays a major role, especially in the emergency caused by the global COVID-19 pandemic. Therefore, we performed homologous and heterologous and heterologous immunizations using DNA vaccine candidates based on the VOCs to develop a safe and effective vaccine strategy based on the prevalent SARS-CoV-2 situation.

In the present study, we evaluated the immunogenicity of the vaccine candidates by processing induced humoral and cellular immune responses in mice vaccinated with 50 μg of the prime-boost DNA vaccine candidates. Based on our prior study on the dose-dependent effects of the full-length SARS-CoV-2 S DNA vaccine candidates, a 50-μg antigen dose most effectively triggered immune responses by showing the highest antibody and NAb titers [23]. Herein, we observed that the mice immunized with DNA vaccine candidates based on the S genotype and Alpha and Beta variants had higher total IgG levels and neutralization antibody responses than the others; the vaccine candidate based on the Alpha variant induced an exceptionally robust and diverse cytokine response. In this study, we discovered that mice vaccinated with the DNA vaccine candidates showed high levels of various cytokines, such as IL-6, IL-13, and IFN-γ, and the Alpha candidate induced tumor necrosis factor-α (TNF-α) production. These cytokines are associated with essential functions that activate and differentiate inflammatory and immune responses in patients with COVID-19 [24,25] and comprise integrated immune signatures in patients infected with SARS-CoV-2 [26]. In mice that received homologous vaccination with Alpha, elevated levels of IL-6 and IL-13 were associated with reduced angiotensin-converting enzyme-2 expression and increased host cell entry of SARS-CoV-2 in airway epithelial cells, particularly in the nasal and bronchial epithelium [27–30]. Specifically, the type 2 cytokine IL-13 is known as the central mediator of lung inflammation caused by respiratory allergies and less severe COVID-19 infection [26,27,31]. IFN-γ is known to be an essential antiviral and antimicrobial cytokine. It is upregulated both locally and systemically in the mucosa and involved in regulating the differentiation of CD4 T cells into Th1 effectors, which moderate the cellular immune system [29,32–34]. In particular, IFN-γ is a treatment target in many diseases, including COVID-19. Several scientific studies have demonstrated that decreased circulating IFN-γ levels are a risk factor for lung fibrosis in COVID-19 infection [35,36]. In addition, mice vaccinated with the Alpha candidate had increased levels of TNF-α compared to levels in the other groups. The pro-inflammatory cytokine TNF-α plays a key role in protecting against infectious pathogens that cause acute and chronic inflammation by inhibiting the replication of the infectious agent via activation of innate immune responses, promotion of cellular apoptosis and proliferation, and simultaneous stimulation of the production of other chemokines and cytokines [37–39]. Moreover, TNF-α is crucial for pathological processes, including cell survival, differentiation, and proliferation, and it promotes the recruitment of macrophages, dendritic cells, and neutrophils to areas invaded by viral contagions in order to control and clear the contagions [37–40]. Numerous studies have investigated the cytokine release syndrome, which is commonly discovered in patients with SARS-CoV-2 and is characterized by excessive secretion of TNF-α, IL-6, and other inflammatory cytokines [37,41]. Other studies have indicated that TNF-α may be a major cytokine that plays a protective role as part of the host response especially in acute viral myocarditis, hepatitis B virus (HBV), and respiratory syncytial virus infection [42–45]. TNF-α modulation may result in increased rates of protein synthesis and reduced rates of protein degradation in adult cardiac myocytes [43]. TNF-α also exerts antiviral activity by promoting the infiltration of leukocytes into affected areas in order to eliminate infectious factors [44]. Moreover, TNF-α may enhance the heart’s ability to contract and induce moderate compensatory hypertrophy and normal tissue homeostasis in cardiac myocytes under stressful conditions [43,44]. Furthermore, TNF-α and IFN-γ produced by HBV-specific cytotoxic T cells inhibit the expression and replication of HBV [44,45]. Therefore, understanding the roles of IL-6, IL-13, IFN-γ, and TNF-α is essential for developing an effective vaccine and treatment against SARS-CoV-2 and other respiratory diseases.

Since the beginning of the COVID-19 pandemic, the role of T cell activation has been extensively studied in clinical trials and experimental models. Secretory Th1 cells promote cellular immune responses and are required for host defense against intracellular pathogens. Th2 cells mediate humoral responses to activate and maintain the antibody reaction against bacteria, allergens, and toxins [34,36,46,47]. In this study, we demonstrated that mice vaccinated with S/S and S/Alpha showed significantly increased levels of Th1 cell-secreted cytokines, such as IFN-γ, and Th2 cytokines, such as IL-6 and IL-13. In particular, we discovered that high levels of S-specific IgG1, a representative Th2 cytokine, IgG2b, and IgG2c are modulated by Th1 cells in all groups, especially in the S/S and S/Alpha groups. These data suggest that DNA vaccine candidates drive robust Th1 and Th2 responses by maintaining cytokine balance. Therefore, modulating these subsets of Th1 and Th2 reactions plays a prominent role in controlling and treating primary SARS-CoV-2 infection by developing an adaptive immune system.

In this study, mice vaccinated with the S/S and S/Alpha variants showed high levels of total IgG, IgG subtypes, and neutralization antibody responses. Even though it remains unclear whether homologous or heterologous DNA vaccine candidates are superior in eliciting humoral and cellular immune responses against SARS-CoV-2 infections, the results of this study indicate that the DNA vaccine candidates based on the S and Alpha variants might be a more effective approach to enhance immune responses than the other candidates. Vaccination with booster doses, whether homologous or heterologous, requires prolonging and improving the immune response by increasing their protective efficacy against SARS-CoV-2 [48]. Moreover, our data showed similar results regardless of prime-boost vaccination programs, in which mice in the S/S and S/Alpha groups elicited both humoral and cellular immune responses compared with others. This result indicates that the efficacy of the DNA vaccine candidates used in this study was variant-dependent. Further studies are needed to confirm which vaccination strategies are optimal for long-term immunity and which T resident memory cells will contribute to protection after vaccination against SARS-CoV-2.

## References

[1] World Health Organization. WHO coronavirus disease (COVID-19) dashboard. 2023 Sep 13. [Cited 2023 Sep 13]. Available from: https://www.who.int.

[2] Grant MC, Geoghegan L, Arbyn M, Mohammed Z, McGuinness L, Clarke EL, et al. The prevalence of symptoms in 24,410 adults infected by the novel coronavirus (SARS-CoV-2; COVID-19): A systematic review and meta-analysis of 148 studies from 9 countries. PLoS One. 2020;15: e0234765. doi: 10.1371/journal.pone.0234765 32574165 PMC7310678

[3] Hasan DA, Maulud SQ, Jalal PJ, Priyanka, Choudhary OP. SARS-CoV-2 vaccine breakthrough reinfection in a health-care worker of Iraq: A case report. Hum Vaccin Immunother. 2022;18: 2055947. doi: 10.1080/21645515.2022.2055947 35417318 PMC9248933

[4] Lim JME, Hang SK, Hariharaputran S, Chia A, Tan N, Lee ES, et al. A comparative characterization of SARS-CoV-2-specific T cells induced by mRNA or inactive virus COVID-19 vaccines. Cell Rep Med. 2022;3: 100793. doi: 10.1016/j.xcrm.2022.100793 36257326 PMC9534788

[5] Malhotra S, Mani K, Ladha R, Sameer B, Mathur VP, Gupta P, et al. COVID-19 infection, and reinfection, and vaccine effectiveness against symptomatic infection among health care workers in the setting of omicron variant transmission in New Delhi, India. Lancet Reg Health Southeast Asia. 2022;3; 100023. doi: 10.1016/j.lansea.2022.100023 35769163 PMC9167830

[6] Chemaitelly H, Tang P, Hasan MR, AlMukdad S, Yassine HM, Benslimane FM, et al. Waning of BNT162b2 vaccine protection against SARS-CoV-2 infection in Qatar. N Engl J Med. 2021;385: e83. doi: 10.1056/NEJMoa2114114 34614327 PMC8522799

[7] Park A. Here’s How effective the original vaccines are against Omicron. TIME. 2022 Sep 22 [Cited 2023 September 12]. Available from: https://time.com/6215580/original-vaccines-effectiveness-against-omicron/.

[8] Kupferschmidt K. New coronavirus variants could cause more reinfections, require updated vaccines. Science. 2021 Jan 15 [Cited 2023 September 1]. Available from: https://www.science.org/content/article/new-coronavirus-variants-could-cause-more-reinfections-require-updated-vaccines.

[9] Schalk JAC, Mooi FR, Berbers GAM, van Aerts LAGJM, Ovelgönne H, Kimman TG. Preclinical and clinical safety studies on DNA vaccines. Hum Vaccin. 2006;2: 45–53. doi: 10.4161/hv.2.2.2620 17012886

[10] Li L, Saade F, Petrovsky N. The future of human DNA vaccines. J Biotechnol. 2012;162: 171–182. doi: 10.1016/j.jbiotec.2012.08.012 22981627 PMC3511659

[11] Silveira MM, Moreira GMSG, Mendonça M. DNA vaccines against COVID-19: Perspectives and challenges. Life Sci. 2021;267: 118919. doi: 10.1016/j.lfs.2020.118919 33352173 PMC7749647

[12] Gary EN, Weiner DB. DNA vaccines: prime time is now. Curr Opin Immunol. 2020;65: 21–27. doi: 10.1016/j.coi.2020.01.006 32259744 PMC7195337

[13] Saade F, Petrovsky N. Technologies for enhanced efficacy of DNA vaccines. Expert Rev Vaccines. 2012;11: 189–209. doi: 10.1586/erv.11.188 22309668 PMC3293989

[14] Atmar RL, Lyke KE, Deming ME, Jackson LA, Branche AR, El Sahly HM, et al. Homologous and heterologous Covid-19 booster vaccinations. N Engl J Med. 2022;386: 1046–1057. doi: 10.1056/NEJMoa2116414 35081293 PMC8820244

[15] Bertoglio F Fühner V, Ruschig M, Heine PA, Abassi L, Klünemann T, et al. A SARS- CoV-2 neutralizing antibody selected from COVID-19 patients binds to the ACE2- RBD interface and is tolerant to most known RBD mutations. Cell Rep. 2021;36. doi: 10.1016/j.celrep.2021.109433 PMC8260561 34273271

[16] Cohen BJ, Audet S, Andrews N, Beeler J. Plaque reduction neutralization test for measles antibodies: Description of a standardised laboratory method for use in immunogenicity studies of aerosol vaccination. Vaccine. 2007;26. doi: 10.1016/j.vaccine.2007.10.046 18063236

[17] Miyah Y, Benjelloun M, Lairini S, Lahrichi A. COVID-19 impact on public health, environment, human psychology, global socioeconomy, and education. Sci. World. J. 2022;2022: 5578284. doi: 10.1155/2022/5578284 35069037 PMC8767375

[18] Lu S. Heterologous prime-boost vaccination. Curr Opin Immunol. 2009;21: 346–351. doi: 10.1016/j.coi.2009.05.016 19500964 PMC3743086

[19] Peng S, Qiu J, Yang A, Yang B, Jeang J, Wang JW, et al. Optimization of heterologous DNA-prime, protein boost regimens and site of vaccination to enhance therapeutic immunity against human papillomavirus-associated disease. Cell Biosci. 2016;6: 16. doi: 10.1186/s13578-016-0080-z 26918115 PMC4766698

[20] Shukarev G, Callendret B, Luhn K, Douoguih M, EBOVAC1 consortium. A two-dose heterologous prime-boost vaccine regimen eliciting sustained immune responses to Ebola Zaire could support a preventive strategy for future outbreaks. Hum Vaccin Immunother. 2017;13: 266–270. doi: 10.1080/21645515.2017.1264755 27925844 PMC5328205

[21] Hill AV, Reece W, Gothard P, Moorthy V, Roberts M, Flanagan K, et al. DNA-based vaccines for malaria: A heterologous prime-boost immunisation strategy. Dev Biol (Basel). 2000;104: 171–179. 11713817

[22] Coughlan L, Sridhar S, Payne R, Edmans M, Milicic A, Venkatraman N, et al. Heterologous two-dose vaccination with simian adenovirus and poxvirus vectors elicits long-lasting cellular immunity to influenza virus A in healthy adults. EBiomedicine. 2018;29: 146–154. doi: 10.1016/j.ebiom.2018.02.011 29519670 PMC5926543

[23] Lim H, Kim SE, Lee YH, Hwang Y-H, Kim SH, Kim MY, et al. Immunogenicity of candidate SARS-CoV-2 DNA vaccines based on the spike protein. Virology. 2022;573: 118–123. doi: 10.1016/j.virol.2022.06.006 35751974 PMC9185170

[24] Justiz Vaillant AA, Qurie A. Interleukin. In: StatPearls. Treasure Island (Florida). 2022.

[25] Dhar SK, V K, Damodar S, Gujar S, Das M. IL-6 and IL-10 as predictors of disease severity in COVID-19 patients: Results from meta-analysis and regression. Heliyon. 2021;7: e06155. doi: 10.1016/j.heliyon.2021.e06155 33553782 PMC7846230

[26] Ramírez-Martínez G, Jiménez-Álvarez LA, Cruz-Lagunas A, Ignacio-Cortés S, Gómez-García IA, Rodríguez-Reyna TS, et al. Possible role of matrix metalloproteinases and TGF-β in COVID-19 severity and sequelae. J Interferon Cytokine Res. 2022;42: 352–368. doi: 10.1089/jir.2021.0222 35647937 PMC9422783

[27] Donlan AN, Sutherland TE, Marie C, Preissner S, Bradley BT, Carpenter RM, et al. IL-13 is a driver of COVID-19 severity. JCI Insight. 2021;6: e150107. doi: 10.1172/jci.insight.150107 34185704 PMC8410056

[28] Peebles RS Jr. IL-13 protects against SARS-CoV-2? Am J Respir Cell Mol Biol. 2022;66: 351–352. doi: 10.1165/rcmb.2021-0562ED 35085479 PMC8990114

[29] Jackson DJ, Busse WW, Bacharier LB, Kattan M, O’Connor GT, Wood RA, et al. Association of respiratory allergy, asthma, and expression of the SARS-CoV-2 receptor ACE2. J Allergy Clin Immunol. 2020;146: 203–206.e3. doi: 10.1016/j.jaci.2020.04.009 32333915 PMC7175851

[30] Kimura H, Francisco D, Conway M, Martinez FD, Vercelli D, Polverino F, et al. Type 2 inflammation modulates ACE2 and TMPRSS2 in airway epithelial cells. J Allergy Clin Immunol. 2020;146: 80–88.e8. doi: 10.1016/j.jaci.2020.05.004 32422146 PMC7227558

[31] Corren J. Role of interleukin-13 in asthma. Curr Allergy Asthma Rep. 2013;13: 415–420. doi: 10.1007/s11882-013-0373-9 24026573

[32] Schoenborn JR, Wilson CB. Regulation of interferon‐γ during innate and adaptive immune responses. Adv Immunol. 2007;96: 41–101. doi: 10.1016/S0065-2776(07)96002-2 17981204

[33] Heuberger J, Trimpert J, Vladimirova D, Goosmann C, Lin M, Schmuck R, et al. Epithelial response to IFN-γ promotes SARS-CoV-2 infection. EMBO Mol Med. 2021;13: e13191. doi: 10.15252/emmm.202013191 33544398 PMC7995094

[34] Cêtre C, Pierrot C, Cocude C, Lafitte S, Capron A, Capron M, et al. Profiles of Th1 and Th2 cytokines after primary and secondary infection by *Schistosoma mansoni* in the semipermissive rat host. Infect Immun. 1999;67: 2713–2719. doi: 10.1128/IAI.67.6.2713-2719.1999 10338473 PMC96574

[35] Abdel-Hamed EF, Ibrahim MN, Mostafa NEW, Moawad HSF, Elgammal NE, Darwiesh EM, et al. Role of interferon gamma in SARS-CoV-2-positive patients with parasitic infections. Gut Pathog. 2021;13: 29. doi: 10.1186/s13099-021-00427-3 33947467 PMC8096133

[36] Chung NH, Chen YC, Yang SJ, Lin YC, Dou HY, Hui-Ching Wang LHC, et al. Induction of Th1 and Th2 in the protection against SARS-CoV-2 through mucosal delivery of an adenovirus vaccine expressing an engineered spike protein. Vaccine. 2022;40: 574–586. doi: 10.1016/j.vaccine.2021.12.024 34952759 PMC8677488

[37] Zawawi ZM, Kalyanasundram J, Zain RM, Thayan R, Basri DF, Yap WB. Prospective roles of tumor necrosis factor-alpha (TNF-α) in COVID-19: prognosis, therapeutic and management. Int J Mol Sci. 2023;24: 6142. doi: 10.3390/ijms24076142 37047115 PMC10094668

[38] Guo Y, Hu K, Li Y, Lu C, Ling K, Cai C, et al. Targeting TNF-α for COVID-19: recent advanced and controversies. Front Public Health. 2022;10: 833967. doi: 10.3389/fpubh.2022.833967 35223745 PMC8873570

[39] Mehta AK, Gracias DT, Croft M. TNF activity and T cells. Cytokine. 2018; 101: 14–18. doi: 10.1016/j.cyto.2016.08.003 27531077 PMC5305780

[40] Jang DI, Lee AH, Shin HY, Song HR, Park JH, Kang TB, et al. The role of tumor necrosis factor alpha (TNF-α) in autoimmune disease and current TNF-α inhibitors in therapeutics. Int J Mol Sci. 2021;22: 2719. doi: 10.3390/ijms22052719 33800290 PMC7962638

[41] Moore JB, June CH. Cytokine release syndrome in severe COVID-19. Science. 2020;368: 473–474. doi: 10.1126/science.abb8925 32303591

[42] Neuzil KM, Tang YW, Graham BS. Protective role of TNF-alpha in respiratory syncytial virus infection in vitro and in vivo. Am J Med Sci. 1996;311: 201–204. doi: 10.1097/00000441-199605000-00001 8615393

[43] Yokoyama T, Nakano M, Bednarczyk JL, McIntyre BW, Entman M, Mann DL. Tumor necrosis factor-alpha provokes a hypertrophic growth response in adult cardiac myocytes. Circulation. 1997;95: 1247–1252. doi: 10.1161/01.cir.95.5.1247 9054856

[44] Wada H, Saito K, Kanda T, Kobayashi I, Fujii H, Fujigaki S, et al. Tumor necrosis factor-α (TNF-α) plays a protective role in acute viral myocarditis in mice. Circulation. 2001;103: 743–749. doi: 10.1161/01.cir.103.5.743 11156888

[45] Guidotti LG, Ishikawa T, Hobbs MV, Matzke B, Schreiber R, Chisari FV. Intracellular inactivation of the hepatitis B virus by cytotoxic T lymphocytes. Immunity. 1996;4: 25–36. doi: 10.1016/s1074-7613(00)80295-2 8574849

[46] Chaplin DD. Overview of the immune response. J Allergy Clin Immunol. 2010;125: S3–S23. doi: 10.1016/j.jaci.2009.12.980 20176265 PMC2923430

[47] Aleebrahim-Dehkordi E, Molavi B, Mokhtari M, Deravi N, Fathi M, Fazel T, et al. T helper type (Th1/Th2) responses to SARS-CoV-2 and influenza A (H1N1) virus: From cytokines produced to immune responses. Transpl Immunol. 2022;70: 101495. doi: 10.1016/j.trim.2021.101495 34774738 PMC8579696

[48] Lyke KE, Atmar RL, Dominguez Islas C, Posavad CM, Szydlo D, Chourdhury RP, et al. SARS-CoV-2 omicron neutralization after heterologous vaccine boosting. medRxiv 2022.

